# Bone Marrow Mesenchymal Stem Cell-Derived Exosomes Ameliorate Aging-Induced BTB Impairment in Porcine Testes by Activating Autophagy and Inhibiting ROS/NLRP3 Inflammasomes via the AMPK/mTOR Signaling Pathway

**DOI:** 10.3390/antiox13020183

**Published:** 2024-01-31

**Authors:** Yi Zhou, Jiale Yan, Limin Qiao, Jiaqin Zeng, Fuyu Cao, Xihui Sheng, Xiaolong Qi, Cheng Long, Bingying Liu, Xiangguo Wang, Hua Yao, Longfei Xiao

**Affiliations:** 1 Animal Science and Technology College, Beijing University of Agriculture, Beijing 102206, China; 202330311015@bua.edu.cn (Y.Z.); 19334205926@163.com (J.Y.); zengqinqin1021@163.com (J.Z.); caofuyu13264417953@outlook.com (F.C.); shengxihui@126.com (X.S.); buaqxl@126.com (X.Q.); dxfdn666@163.com (C.L.); liuby1226@163.com (B.L.); xiangguo731@163.com (X.W.); 2 Department of Animal Husbandry and Veterinary, Beijing Vocational College of Agriculture, Beijing 102445, China; limingqiao123@126.com

**Keywords:** senescence, blood-testis barrier, BMSC-Exos, autophagy, tight junction

## Abstract

As a pivotal player in spermatogenesis, the blood-testis barrier (BTB) made from junction apparatus coexisting in Sertoli cells (SCs) is impaired with an increase in age and ultimately induces spermatogenic dysfunction or even infertility. It has been corroborated that bone marrow mesenchymal stem cell (BMSC) transplantation can efficiently repair and regenerate the testicular function. As vital mediators of cell-to-cell communication, MSC-derived exosomes (Exos) can directly serve as therapeutic agents for tissue repair and regeneration. However, the therapeutic value of BMSC-Exos in aging-induced BTB damage remains to be confirmed. In this study, we explored that the old porcine testes had defective autophagy, which aggravated BTB disruption in SCs. BMSC-Exos could decrease ROS production and NLRP3 inflammasome activation but enhanced autophagy and tight junction (TJ) function in D-gal-triggered aging porcine SCs and mouse model testes, according to in vitro and in vivo experiments. Furthermore, rapamycin, NAC, MCC950, and IL-1Ra restored the TJ function in D-gal-stimulated aging porcine SCs, while BMSC-Exos’ stimulatory effect on TJ function was inhibited by chloroquine. Moreover, the treatment with BMSC-Exos enhanced autophagy in D-gal-induced aging porcine SCs by means of the AMPK/mTOR signal transduction pathway. These findings uncovered through the present study that BMSC-Exos can enhance the BTB function in aging testes by improving autophagy via the AMPK/mTOR signaling pathway, thereby suppressing ROS production and NLRP3 inflammasome activation.

## 1. Introduction

It is generally believed that the negative effects related to reproduction caused by increased parents’ age are mainly attributable to females. However, there is growing evidence that the increase in male age has a significantly negative impact on spermatogenesis, sperm function, fertilization, pregnancy, and offspring health [1]. Senescence also impacts the entire endocrine system, including the hypothalamic-pituitary-gonadal (HPG) axis, which is critical for the production of hormones such as testosterone and leads to changes in testis physiology as well as fertility status [2]. In addition, it has been demonstrated that aging can attenuate the function of blood-testis barrier (BTB), which is constituted by coexisting tight junction (TJ) between adjacent Sertoli cells (SCs). The prominently lowered expression of TJ-related proteins (Claudin-11, ZO-1, and Occludin) has been detected in natural [3] and iatrogenic ageing (such chronic D-galactose exposure [4]) rodent models, suggesting that aging indeed damages the BTB function in the testes to induce spermatogenesis dysfunction.

Autophagy refers to the dynamic of self-protection and cell defense mechanism that serves as a valid routine to remove hazardous and toxic matters by cells [5]. It has been increasingly evidenced that autophagy in SCs plays an essential role in normal generation of sperms and fertility of males [6]. It has also been recently researched that autophagy is weakened in rat testes during aging [4]. The damaged cells or organelles together with cumulated metabolic wastes may destroy efficient autophagy modulation as the age increases [7]. Oxidative stress is defined as a state of imbalance between excessive oxidant (free) radicals and insufficient degradation of those radicals by antioxidant systems as an in-house defense mechanism [8]. Notably, when an organism becomes senescent, the oxidative stress-induced lipoprotein degeneration on cell membrane and organelle impairment emerge as crucial factors for organ and cell functional decline, while autophagy relieves the impairment attributable to reactive oxygen species (ROS) accumulation during senescence [9,10].

As testified by enormous studies, there is a relationship between excess ROS production in cells and the infertility of males besides testis injury [11,12]. To be more specific, it is also a pivotal player in TJ impairment in SCs [13]. It is argued that NLRP3 inflammasomes can be activated by ROS as a leading triggering factor [14]. As a multiprotein complex with Caspase-1, apoptosis-associated speck-like protein containing a caspase recruitment domain (ASC) and NLRP3 as the constituents, NLRP3 inflammasome not only mediates Caspase-1 activation but also subsequently promotes IL-1*β* plus IL-18 to mature and release [13]. The latest studies have manifested that such typical cytokines may induce or worsen the inflammation in cells or organs due to their functions to mediate inflammatory responses in diversified cells, thus promoting disease progression [15].

Classified as mesoderm-derived stem cells, bone marrow mesenchymal stem cells (BMSCs) have the ability to differentiate into various types of cells involving muscle cells, osteoblasts, adipocytes, and chondrocytes. A growing number of studies have demonstrated that transplanted MSCs could increase the reproductive ability during natural aging or in modeling of senescent animals by virtue of agents [16,17]. A few investigations conducted in recent years have demonstrated that BMSCs can secrete exosomes (Exos), which are special membranous vesicles in nano-size possessing similar functions to MSCs [16]. Being small membrane-bound vesicles with a diameter of 30–100 nm, Exos can bind to the recipient cell membrane by virtue of internal membrane contents and deliver a variety of biomolecules (nucleic acids, proteins, lipids, etc.). Although BMSC-derived Exos (BMSC-Exos) have been considered as a promising therapeutic tool for resisting aging [18], the mechanism by which Exos improve the prognosis has not been entirely understood. Thus, the beneficial effect of BMSC-Exos on aging-induced TJ impairment was explored in the present study through both in vitro experiment and mouse model. The data obtained thereof will provide useful information for developing a new therapeutic approach to improve fertility in elder boars.

## 2. Materials and Methods

### 2.1. Collection of Porcine Testes

A total of 3 young (2–3 years old) and 3 old (5–6 years old) Landrace pigs were selected from the local station for testis collection. Samples (size: approximately 3 × 3 cm^2^) acquired from the middle testes were directly cryopreserved (−80 °C) by liquid N_2_ for subsequent extraction of proteins as well as RNAs. The remaining testes were subjected to fixation using glutaraldehyde (2.5%) or paraformaldehyde (4%) for histological, immunohistochemical, and echocardiographic analyses.

### 2.2. Porcine BMSC Separation, Character Determination, and Differentiation

After flushing with DMEM, the bone marrow acquired from the tibia and femur of 1-month-old Landrace boars was separated through 5 min of 800× *g* centrifugation. Next, the sediments produced underwent inoculation into DMEM (1 × 10^5^ cells/cm^2^) composed of fetal bovine serum (FBS, 10%, Hyclone Laboratory, Logan, UT, USA) mixed with penicillin (100 U/mL) and streptomycin (100 μg/mL) under a humidified atmosphere (37 °C) containing 5% CO_2_. After initial plating, the medium replacement was conducted every 3–4 d. The cells with about 80–90% confluence were passaged for further expansion. Finally, CD29, CD44, and CD45 were selected for characterization using immunofluorescence analysis, so as to detect the classical biomarkers of BMSCs.

The multipotent differentiation potential from porcine into osteoblasts and adipocytes was evaluated. Adipogenic or osteogenic differentiation complete media provided by Cyagen Biosciences (Suzhou, China) were utilized to replace the culture medium of BMSCs passaged to the 3rd generation. Subsequent to 14-day differentiation culture induction, intracellular lipids together with calcium were evaluated for accumulation using oil red O staining plus alizarin red staining (Sigma-Aldrich, St. Louis, MO, USA), respectively.

### 2.3. Porcine SC Segregation and Culture

Normal Landrace boars (1 month old in age) were chosen to obtain the testicular tissues, followed by washing in streptomycin (100 mg/mL) + penicillin (100 IU/mL) added three times into phosphate buffer saline (PBS). Later, isolation and cultivation of SCs were performed in accordance with a slightly modified previous method. Under sterile conditions, the culture medium was used for rinsing of every testis, with the tunica albuginea discarded. The separated testicular parenchyma was divided into sections followed by 15 min of digestion using collagenase type IV (1 mg/mL) at 37 °C. After washing three times in PBS, the convoluted seminiferous tubules were collected under a stereo microscope, followed by an additional 30 min of tissue digestion using collagenase type IV. Next, the cell pellets subjected to 5 min 1000× *g* centrifugation plus three times of culture medium rinsing were resuspended via 10% FBS-containing DMEM in a humidified incubator under 5% CO_2_ + 95% air at 37 °C. Being cultured for 72 h, hypotonic Tris-HCl solution (20 mM, pH 7.4) was applied to treat the cells, from which residual germ cells were eliminated through 2 min of gentle shaking, followed by discarding of Tris-HCl solution. Lastly, immunofluorescence analysis was adopted for characterization with SOX9, so as to measure the classical biomarkers of SCs.

With the confluence reaching 80%, a 6-well plate cell seeding was implemented (density: nearly 1.5 × 10^5^ cells/well) for 24 h of 37 °C cultivation using the humidified incubator under 5% CO_2_ plus 95% O_2_.

### 2.4. Purification and Identification of Exos

Cell Resource Center of Shanghai Institutes for Biological Sciences, Chinese Academy of Sciences (Shanghai, China) was the supplier of murine BMSCs. Following overnight inoculation in 25 cm^2^ culture bottles to realize 80% confluence before use, porcine and murine BMSCs were washed 3 times in PBS in addition to 24 h of serum-free DMEM culture. Exo extraction from the culture media was implemented following the recommendations offered by the manufacturer (BB-3901, Shanghai Bestbio Biotechnology Co., Ltd., Shanghai, China).

The porcine BMSC-Exos were resuspended in 30 μL of PBS, from which specimens (10 μL) were loaded onto a copper mesh for 1 min, and then filter paper was employed to absorb the liquid. Later, the copper mesh was reacted for 1 min in uranyl acetate (phosphotungstic acid, 10 μL), from which the filter paper was used for liquid elimination. Subsequent to room-temperature drying for several minutes, electron microscopy was adopted for imaging (80 kV) to examine the specimens.

### 2.5. PKH26 Staining for Exos

BMSC-Exos received 15 min of PKH26 (Sigma) labeling in the dark (37 °C) and were washed three times in PBS prior to 10,000× *g* and 4 °C centrifugation for 2 h. Later, the prepared SCs were co-cultured for 6 h with the labeled Exos (10 μg/mL). Next, DAPI (C1002, Beyotime, Nanjing, China) counterstaining of the cells was accomplished followed by washing one time in PBS to identify their nuclei. The uptake of MSC-Exos by SCs was observed under a fluorescence microscope.

### 2.6. Cell Treatment

Prior to experiments, serum-free DMEM was applied to culture porcine SCs for 12 h which underwent 48 h of D-galactose (D-gal, 12.5, 25 or 50 g/L in final concentrations for aging induction, HY-N0210, Med Chem Express, Monmouth Junction, NJ, USA), and/or BMSC-Exos (20 μg/mL) processing. For inhibition experiments, 2 h of cell incubation was conducted prior to treatment with or without the supplement of autophagy inducer rapamycin (Rapa, HY-N0210, 200 nM), ROS scavenger acetylcysteine (NAC, HY-B0215, 5 mM), autophagy inhibitor chloroquine (CQ, HY-17589A, 50 μM), AMPK inhibitor Compound C (CC, HY-13418A, 10 μM), NLRP3 inhibitor MCC950 (HY-12815, 10 μM), and IL-1 receptor antagonist (IL-1Ra, HY-P72566, 20 ng/mL) offered by Med Chem Express.

### 2.7. Measurement of Autophagic Flux

Based on the manufacturer’s instructions, the mCherry-GFP-LC3 reporter plasmid (C3011, 1 μL/mL) provided by Beyotime (Nanjing, China) was selected for SC transfection to determine the autophagic flux. Thereafter, the cells underwent grouping and processing through the aforementioned methods. Fluorescence microscopy was performed to observe the cell images.

### 2.8. Senescence-Associated β-Galactosidase (SA-β-Gal) Staining

The SA-β-gal staining kit purchased from Beyotime (C0602, Nanjing, China) was utilized to implement SA-β-gal staining in accordance with the protocol formulated by the manufacturer. The SA-β-gal-positive cells were stained blue. Finally, an optical microscope (Olympus-DP73, Tokyo, Japan) was employed to count positive cells.

### 2.9. ROS and Antioxidant Assessment

The GSH assay kit (S0073, Beyotime) was adopted to examine the SCs of the GSH level according to the manufacturer’s protocol. By reference to the manufacturer’s instructions, DCFH-DA (S0033, Beyotime) was used for total ROS level measurement. SCs (5000 cells/well at concentration) were plated in a 96-well microplate and processed as indicated. Next, the DCFH-DA (10 μmol/L)-loaded cells were placed for 30 min away from light (37 °C) and gently cleaned 3 times in PBS. The fluorescence microscope (Nikon, Tokyo, Japan) together with a microplate reader (Gemini XPS, Molecular Devices, Gothenburg, Sweden) were employed to detect total ROS for the fluorescence intensities.

### 2.10. IL-1β Determination

Enzyme linked immunosorbent assay (ELISA) was executed to measure the culture medium for IL-1*β* concentration as per the detailed procedures described in Porcine IL-1*β* ELISA Kit (JL21874, Jianglaibio, Shanghai, China). Afterward, all samples experienced duplicate measurement to obtain the 450 nm absorbance, and the values of negative controls (sample-free blanks) were subtracted. A total of 1 pg/mL IL-1*β* was set as the minimum detectable concentration.

### 2.11. Animal Grouping and Age Modeling

The processing of all experimental animals was accomplished in compliance with relevant regulations published by the China Council on Animal Care, with the procedures all accomplished by reference to the Guidelines of the Animal Ethics Committee of Beijing University of Agriculture [Permit No.: SYXK(JING)2021-0001].

A total of 40 ICR male mice aged 7–8 weeks old provided by Beijing Vital River Laboratory Animal Technology Co., Ltd. (Beijing, China) were grown in a controlled humid (40–70%) animal house with a 12 h dark/light cycle at (20–25 °C). The mice were subjected to 1-week acclimatization with free access to water in addition to food throughout the study, followed by allocation into four groups: D-gal group (*n* = 10), BMSC group (*n* = 10), control group (*n* = 10), and BMSC-Exos group (*n* = 10). D-gal (200 mg/kg/day) was subcutaneously injected into the mice from the D-gal group daily for 60 d, while saline was administered in an equal volume to the control group for 60 d. On days 30 and 45, the therapeutic groups were infused with 100 μg BMSC-Exos in addition to 1 × 10^6^ BMSCs from the tail vein.

On the last day, with general anesthesia (induced by 150 mg/kg pentobarbital sodium injected intraperitoneally) achieved, all mice were killed. The left testis was preserved at −80 °C immediately following excision to receive biochemical analysis, whereas the right one was fixed in 4% paraformaldehyde or 2.5% glutaraldehyde for histological, immunohistochemical, and echocardiographic analyses.

### 2.12. Western Blotting Analysis

After collection and ice-cold PBS washing, the tissues and cells were treated with ice-cold PMSF (1 mM)-containing RIPA lysis buffer. Western blotting assay was conducted as previously described [4]. The primary antibodies against p-AMPK (Ser485) (1:1000 dilution; #2537; Cell Signaling Technology, Danvers, MA, USA), AMPK (Ser485) (1:1000 dilution; #2532; Cell Signaling Technology), p-ERK1/2 (Thr202/Tyr204) (1:1000 dilution; #9101; Cell Signaling Technology), ERK1/2 (1:1000 dilution; #9102; Cell Signaling Technology), p-AKT (Ser473) (1:1000 dilution; #4060; Cell Signaling Technology), AKT (1:1000 dilution; #4691; Cell Signaling Technology), p-mTOR (Ser2448) (1:1000 dilution; #5536; Cell Signaling Technology), mTOR (1:1000 dilution; #2983; Cell Signaling Technology), ZO-1 (1:1000 dilution; bs-1329R; Bioss, Woburn, MA, USA), Occludin (1:1000 dilution; bs-10011R; Bioss), Claudin-11 (1:1000 dilution; bs-21509R; Bioss), Beclin-1 (1:1000 dilution; 11306-1-AP; Proteintech, Rosemont, IL, USA), LC3 (1:1000 dilution; 14600-1-AP; Proteintech), NLRP3 (1:1000 dilution; 19771-1-AP; Proteintech), ASC (1:1000 dilution; 10500-1-AP; Proteintech), Caspase-1 (1:1000 dilution; 22915-1-AP; Proteintech), IL-1*β* (1:1000 dilution; #12703; Cell Signaling Technology), and *β*-actin (1:3,000 dilution; bs-0061R; Bioss) were utilized. The HRP-conjugated goat anti-rabbit secondary antibody (diluted at 1:3000, bs-0295-HRP; Bioss) was utilized. ECL solution was used for band examination, and the ImageJ 1.44p was adopted for signal quantification.

### 2.13. Histological Analysis

The porcine and murine testicular tissues embedded in paraffin were sliced into 4 μm sections and subjected to hematoxylin-eosin staining. The optical microscope (Olympus-DP73, Tokyo, Japan) was utilized to evaluate the dynamic changes for histological analysis of the testes.

### 2.14. Immunohistochemical Staining

Immunohistochemical staining was conducted based on the procedures in our previous report [4]. Specifically, the overnight incubation (4 °C) of testicular tissue sections was executed by virtue of the rabbit polyclonal antibody against ZO-1 (diluted at 1:100; bs-1329R; Bioss) or LC3 (1:100 dilution; 14600-1-AP, Proteintech) in combination with the mouse monoclonal SOX9 antibody (diluted at 1:100; ab76997; abcam, an SC-specific marker). Later, the tissue sections, washed 3 times in PBS, were cultured for 45 min using the FITC-coupled goat anti-rabbit IgG (H+L) antibody (1:200 dilution; HS111, TransGen, Beijing, China) and the PE-labeled goat anti-mouse IgG (H+L) antibody (1:200 dilution; HS221, TransGen, Beijing, China). Lastly, the cell nuclei received DAPI staining based on the previously described methods, followed by observation and photography of the sections under the Olympus-DP73 optical microscope (Tokyo, Japan).

### 2.15. Evaluation of Oxidative Stress in Testes

The level of lipid peroxidation marker, MDA, in addition to the activity of enzymatic antioxidants, SOD and CAT, were examined to appraise the oxidative stress in murine testes in accordance with the instructions of commercially available kits for MDA (A003-1), SOD (A001-1), and CAT (A007-1) (Jiancheng Bioengineering Institute, Nanjing, China).

### 2.16. Transmission Electron Microscopy (TEM)

The 2.5% glutaraldehyde-fixed testicular tissues received 1 h of a third fixation (4 °C) in osmium tetroxide (1%), prior to rehydration, embedding, slicing, and uranyl acetate + citrate double staining. Finally, the transmission electron microscope (Hitachi H-7500, Hitachi Ltd., Tokyo, Japan) was applied for the ultrastructure observation of the TJs formed by adjacent SCs plus autophagy monitoring in SCs.

### 2.17. Data Analysis

Data are shown as the mean ± SD, and results were analyzed using GraphPad Prism 9 software (GraphPad Software, San Diego, CA, USA). Normal distribution data were analyzed by unpaired Student’s *t*-test for comparisons between two groups or by one-way ANOVA with a Student–Newman–Keuls test for pairwise comparisons between three or more groups. A non-parametric test was used when data were not normally distributed. Data were considered statistically significant at *p* value < 0.05.

## 3. Results

### 3.1. Autophagy Defect and BTB Dysfunction in Old Porcine Testes

**Figure 1:**
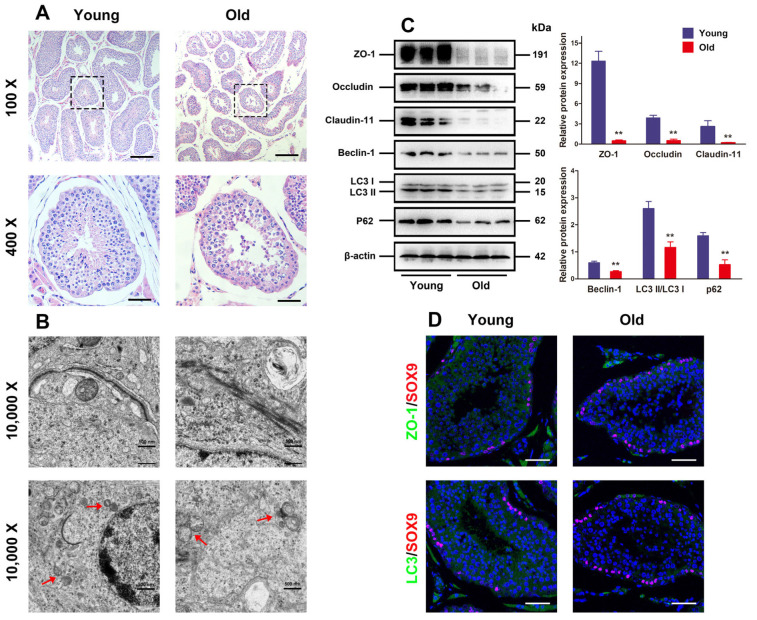
Autophagy defect and BTB dysfunction in old porcine testes. (**A**) The histological variations in the testes are appraised with HE staining. The changes in seminiferous tubules (original magnification = 100×, scale bar = 200 μm) are presented in the upper panels. The magnified images of the boxed areas are exhibited in the lower panels (original magnification set at 400×, scale bar = 50 μm). (**B**) The ultrastructure of autophagy and TJ function in porcine SCs is observed by TEM (original magnification = 10,000×, scale bar = 500 nm). (**C**) The relative expressions of Claudin-11, ZO-1, Occludin, Beclin-1, and LC3 proteins in testicular tissues are determined by means of Western blotting. (**D**) Representative images of immunofluorescence for localization of ZO-1 and LC3 (green) by virtue of SOX9 (red) in the testes are obtained. With β-actin as the loading control, values are expressed by mean ± SD. ** *p* < 0.01 vs. the young group.

First, the histological changes in young and old porcine testes were assessed. It was illustrated in Figure 1A that the testicular tissues of young boars were well organized, with intact seminiferous tubules exhibiting tight and orderly arrangement of spermatogenic cells, and spermatids and spermatozoa were observed in the lumen. Nevertheless, old porcine testes had partially thinned seminiferous epithelium, reduced number of luminal mature sperms, and spermatogenic cells in contrast to the young ones, suggesting that the testes suffer from severe atrophy. Given the pivotal function of testicular TJs formed by adjacent SCs for spermatogenesis, their changes in seminiferous epithelium from the aspects of autophagy and ultrastructure were observed under the TEM. Numerous autophagic vesicles existed in SC cytoplasm of the young porcine testes, with abundant organelles. As for the old porcine testes, the number of autophagic vesicles in the SC cytoplasm was slightly reduced, with visible swollen endoplasmic reticulum, vacuoles, and disorderly arranged mitochondria. Moreover, the tissue sections made from young porcine testes were displayed as clear and integrate the TJ ultrastructure formed by adjacent SCs, while the BTB was progressively disassembled in old porcine testes.

Given the major roles of Claudin-11, ZO-1, and Occludin as proteins involved in the formation of TJ function, Western blotting analysis was carried out to detect their expressions and the expressions of autophagy-related markers LC3 and Beclin-1. The results indicated that the old porcine testes had significantly lower TJ-related proteins and autophagy-related markers than the young ones (Figure 1C, *p* < 0.01). Moreover, the localization of ZO-1 and LC3 proteins evaluated by immunofluorescence analysis manifested that the expressions of ZO-1 and LC3 increased remarkably in the old porcine testes in contrast to the young ones (Figure 1D). These findings suggest that autophagy and BTB function are attenuated with aging.

### 3.2. Alteration of Autophagy Affected TJ Function in D-Gal-Induced Aging Porcine SCs

**Figure 2:**
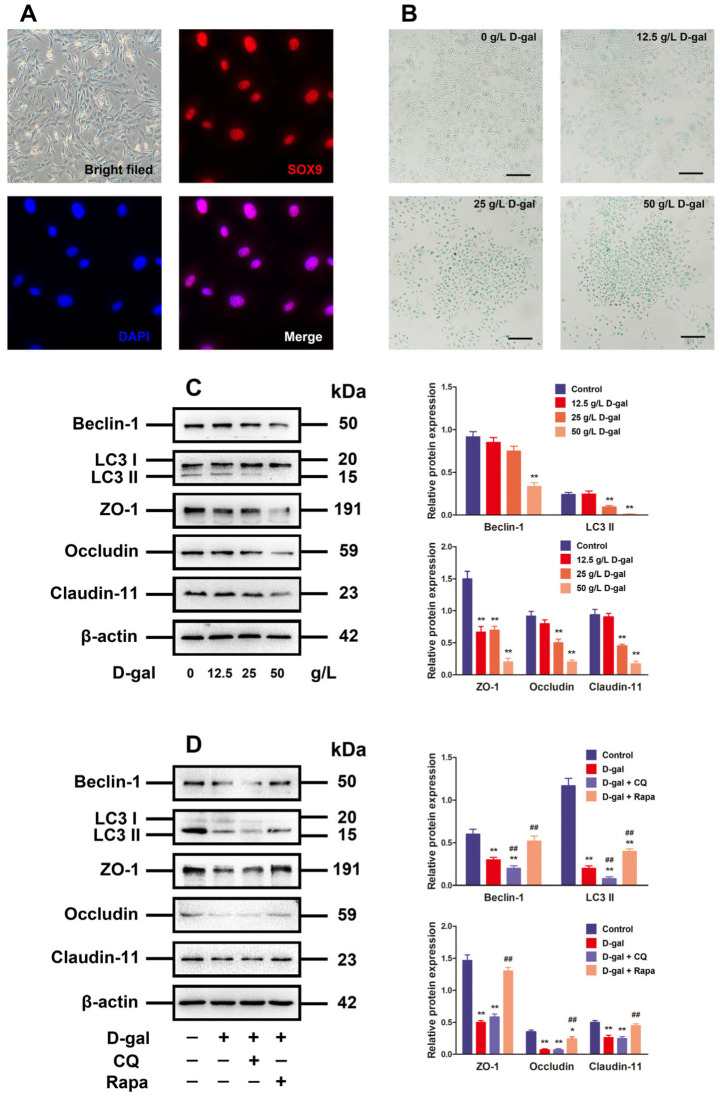
Alteration of autophagy affected TJ function in D-gal-induced aging porcine SCs. (**A**) The SCs are identified via morphological observation and immunofluorescence analysis with SOX9. (**B**) The D-gal-induced SC senescence is determined by SA-β-gal staining (original magnification: 100×, scale bar = 200 μm). (**C**) SCs are treated with 0, 12.5, 20, and 50 g/L D-gal for 48 h. Western blotting is performed on Beclin-1, LC3, ZO-1, Occludin, and Claudin-11 proteins. (**D**) SCs are subjected to 48 h of treatment with D-gal and/or CQ plus rapamycin. Beclin-1, LC3, ZO-1, Occludin, and Claudin-11 proteins are analyzed through Western blotting, with β-actin as the loading control. The mean ± SD is applied to exhibit the values; * *p* < 0.05 and ** *p* < 0.01 vs. the control group; ^##^ *p* < 0.01 vs. the D-gal treatment group.

The primary culture of porcine SCs aimed to validate the mechanism of aging in impacting the BTB disruption through its effect on SCs by regulating autophagy. The porcine SCs were round or square in shape, whose nuclei were red as indicated by the anti-SOX9 antibody staining (Figure 2A). The chronic D-gal exposure for premature aging stimulation is able to induce similar characteristics to natural aging [4,19,20]. As a result, aging was simulated in the D-gal-treated SCs in this research. Following 48 h of SC processing under D-gal (0, 12.5, 25, and 50 g/L at concentration), the results of β-gal staining revealed that the high-dose D-gal treatment group was detected with more β-gal-positive cells (blue stained) (Figure 2B). Moreover, D-gal was able to decrease autophagy-related markers (LC3 and Beclin-1) in addition to TJ-related proteins (Claudin-11, ZO-1, and Occludin) in a dose-dependent manner, whereas 50 g/L D-gal prominently reduce the expressions of such proteins compared to the control group (Figure 2C, *p* < 0.01).

To investigate the relationship between autophagy and TJ function in aging models, the D-gal-induced aging porcine SCs were treated with CQ (the autophagy inhibitor) or rapamycin (the autophagy inducer) for 48 h. With the autophagy in D-gal-induced aging porcine SCs restrained, LC3 and Beclin-1 protein declined significantly in contrast to those in D-gal groups (*p* < 0.01), but there were no significant impacts on the expressions of TJ-related proteins. On the contrary, the activated autophagy blocked the inhibitory effect of D-gal on autophagy-related markers and TJ-related proteins in terms of expression (Figure 2D). The above results imply that D-gal disrupts the TJ function through restricting the autophagic flux.

### 3.3. Characterization of BMSC-Exos and Exos Inhibited D-Gal-Induced Autophagy Defect and TJ Dysfunction in Porcine SCs

**Figure 3:**
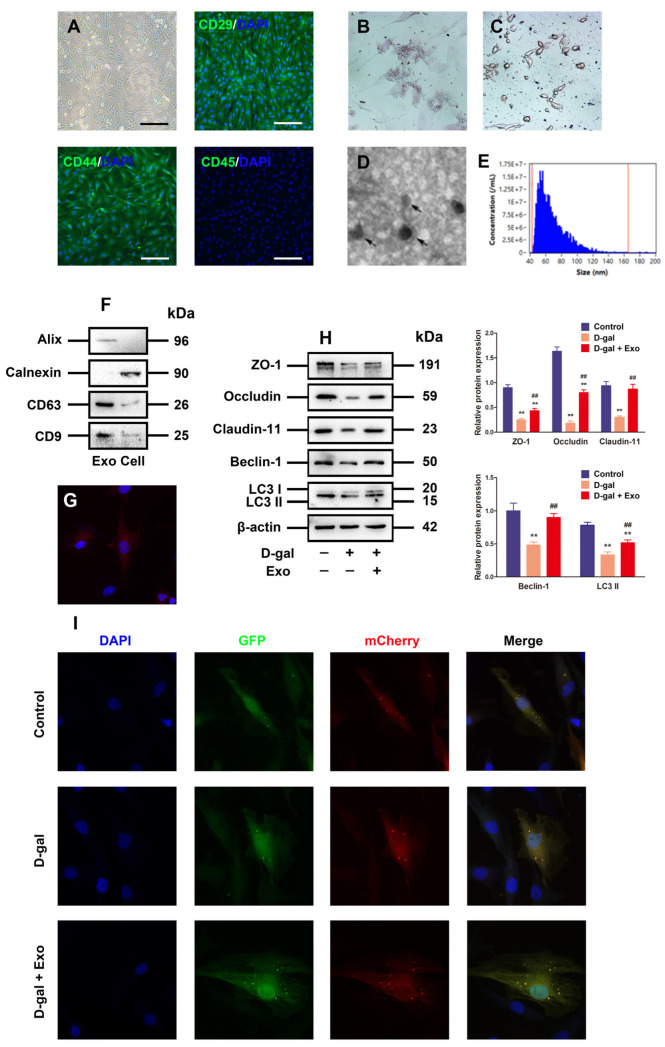
Characterization of BMSC-Exos and Exos inhibits D-gal-induced autophagy defect and TJ dysfunction in porcine SCs. (**A**) Morphological observation and immunofluorescence analysis of CD29, CD44, and CD45 are identified from the porcine BMSCs (original magnification = 100×, scale bar = 200 μm). (**B**,**C**) Phenotypes of porcine BMSCs differentiating into adipocytes and osteocytes are determined by light microscopy. (**D**) TEM is adopted for Exo analysis, scale bar = 500 nm. (**E**) The diameters of Exos are measured using nanoparticle trafficking analysis (NTA). (**F**) Western blotting is employed to detect the expressions of positive markers (Alix, CD63, and CD9) and negative markers (Calnexin) in Exos. (**G**) Fluorescence image of PKH67-labeled Exos taken up by porcine SCs is presented. (**H**) ZO-1, Occludin, Claudin-11, Beclin-1, and LC3 proteins are examined through Western blotting provided with the loading control of β-actin. (**I**) Autophagy is visually observed via transfection of SCs with mCherry-GFP-LC3 adenovirus and quantitation of autolysosomes. The mean ± SEM is selected to present the values; ** p < 0.01 vs. the control group; ^##^ *p* < 0.01 vs. the D-gal treatment group.

The BMSCs obtained from the porcine bone marrow were observed as fibrocyte-like adherent cells in a long spindle shape, and BMSCs highly expressed CD29 and CD44, but they were negative for CD45 according to immunofluorescence analysis (Figure 3A). Additionally, the BMSCs showed alizarin red-positive calcium nodules (Figure 3B) or oil red O-positive lipid droplets (Figure 3C) following culture with osteogenic and adipogenic induction medium, respectively, demonstrating that the BMSCs acquired therein possess preferable characterization and identification.

The conditioned BMSC medium was harvested for centrifugation to extract Exos from porcine BMSCs. Then, the previously described Exo characteristics were set as the basis to distinguish the isolated particles from the aspects of morphology and phenotype. Firstly, the BMSC-derived particles underwent direct visualization under the TEM to obtain their morphology, where round or elliptical nanovesicles were identified based on the particles, with a double-layer membrane structure (Figure 3D). Secondly, nanoparticle tracking analysis (NTA, NanoSight, Malvern, UK) was executed to measure the distribution of particle size, uncovering that the particle size ranged from 54 nm to 148 nm, averaged on 84 nm (Figure 3E). Thirdly, Western blotting was employed to evaluate the Exo-specific markers CD9, Alix, and CD63, with high expressions in the particles, but the BMSC marker protein Calnexin was negatively expressed (Figure 3F). Therefore, the aforementioned analysis of properties showed that the isolated BMSC-derived particles were identified as Exos in this study.

To investigate whether BMSC-Exos can be absorbed by alveolar macrophages, Exos were labeled with PKH26. After the 12 h co-incubation of labeled Exos stained with PKH26 and porcine SCs, the Exo pellets in the cytoplasm manifested intensive red fluorescence (Figure 3G), indicating that Exos are absorbed by porcine SCs. Moreover, the Western blotting analysis results uncovered that BMSC-Exos obstructed the inhibitory effect of D-gal on the expressions of autophagy-related markers and TJ-related proteins (Figure 3H). Furthermore, it was found through mCherry-GFP-LC3 fluorescence analysis that the formations of autophagosome and autolysosome punctum declined in SCs exposed to 50 g/L D-gal in contrast to those in the control group, while they were effectively attenuated by the BMSC-Exo treatment under D-gal induction (Figure 3I). Therefore, our results confirm that BMSC-Exos can effectively promote the autophagic flux and TJ function of porcine SCs exposed to D-gal.

### 3.4. BMSC-Exos Recovered TJ Function through Promoting Autophagic Flux to Inhibit NLRP3 Inflammasome Activation Plus ROS Generation in D-Gal-Induced Aging Porcine SCs

**Figure 4:**
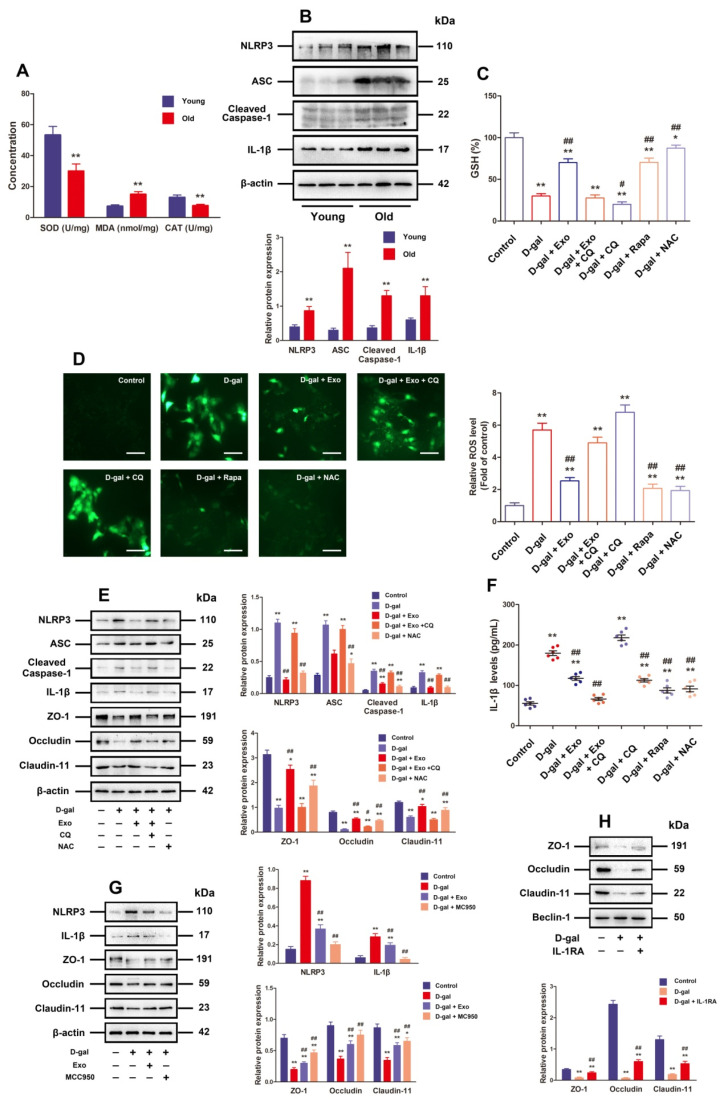
BMSC-Exos recover TJ function by increasing autophagic flux, so as to inhibit ROS generation and NLRP3 inflammasome activation in D-gal-induced aging porcine SCs. (**A**) SOD, MDA, and CAT content in young and old porcine testes is detected. (**B**) Western blotting is carried out to measure the relative protein expressions of NLRP3, ASC, Cleaved-Caspase-1, and IL-1*β* in porcine testes. (**C**) GSH level is calculated (*n* = 6). (**D**) Total ROS levels are detected with DCFH-DA (scale bar = 50 μm). (**E**) SCs are treated with D-gal (50 g/L), CQ (50 μM), NAC (5 mM), and/or BMSC-Exos (20 μg/mL) for 48 h. The protein expressions of NLRP3, ASC, Cleaved-Caspase-1, IL-1*β*, ZO-1, Occludin, and Claudin-11 are researched by means of Western blotting. (**F**) IL-1β secretion is investigated by ELISA by virtue of cell culture supernatant fractions (*n* = 6). (**G**) SCs are treated with D-gal (50 g/L), MCC950 (10 μM), and/or BMSC-Exos (20 μg/mL) for 48 h. NLRP3, IL-1*β*, ZO-1, Occludin, and Claudin-11 are researched for their protein expressions via Western blotting. (**H**) SCs undergo 48 h of D-gal (50 g/L) and/or IL-1Ra (20 ng/mL) treatment. Western blotting is executed to probe into ZO-1, Occludin, and Claudin-11 for protein expressions, during which β-actin serves as the loading control, and the values are expressed by mean ± SD; * *p* < 0.05 and ** *p* < 0.01 vs. the control group; ^#^ *p* < 0.05 and ^##^ *p* < 0.01 vs. the D-gal treatment group.

ROS possesses the crucial function of triggering NLRP3 inflammasome activation, and the latter can cause barrier function disorder as well. According to Figure 4A, the old porcine testes exhibited evidently lower CAT and SOD content (*p* < 0.01) and prominently higher MDA content than the young porcine testes (*p* < 0.01). Moreover, Western blotting results showed that the old porcine testes exhibited clearly increased protein expressions concerning ASC, Cleaved-Caspase-1, NLRP3, and IL-1*β* compared with the young ones (Figure 4B, *p* < 0.01). These results denote that the old porcine testes are undergoing oxidative stress and inflammation.

Furthermore, the results of in vitro experiment revealed notably lowered GSH and higher ROS levels when SCs were exposed to 50 g/L D-gal in contrast to those in the control group, but the BMSC-Exos, rapamycin, and NAC processing were able to effectively reduce ROS and increase GSH levels in SCs under D-gal induction, while the effect of BMSC-Exos was repressed by CQ (Figure 4C,D). In addition, Western blotting results presented that compared with those in the control group, the protein expressions of NLRP3, Cleaved-Caspase-1, ASC, and IL-1*β* together with IL-1*β* release were evidently increased in SCs processed with 50 g/L D-gal, while BMSC-Exos and NAC treatment could not only effectively attenuate NLRP3 inflammasome-related protein expressions and IL-1*β* release but also promote TJ-related proteins (Claudin-11, ZO-1, and Occludin) in terms of expression in D-gal-induced aging SCs, whereas such effects of BMSC-Exos were blocked by CQ (Figure 4E,F). These data corroborate that autophagy participates in the anti-oxidation and NLRP3 inflammasome activation of BMSC-Exos in D-gal-induced aging SCs.

IL-1Ra combined with MCC950 (a selective inhibitor of NLRP3) was selected to appraise whether the TJ dysfunction is attributable to D-gal-triggered NLRP3 activation besides increased IL-1*β* release. The facilitation of D-gal for NLRP3 and IL-1 protein expressions was inhibited by MCC950, and both MCC950 and IL-1Ra were capable of effectively facilitating the expressions of TJ-related proteins in porcine SCs exposed to D-gal (Figure 4G,H). Taken together, BMSC-Exos can recover TJ function via enhancing the autophagic flux to inhibit ROS generation and NLRP3 inflammasome activation in D-gal-induced aging porcine SCs.

### 3.5. BMSC-Exos Regulated Autophagy and TJ Function through the AMPK/mTOR Signaling Pathway

**Figure 5:**
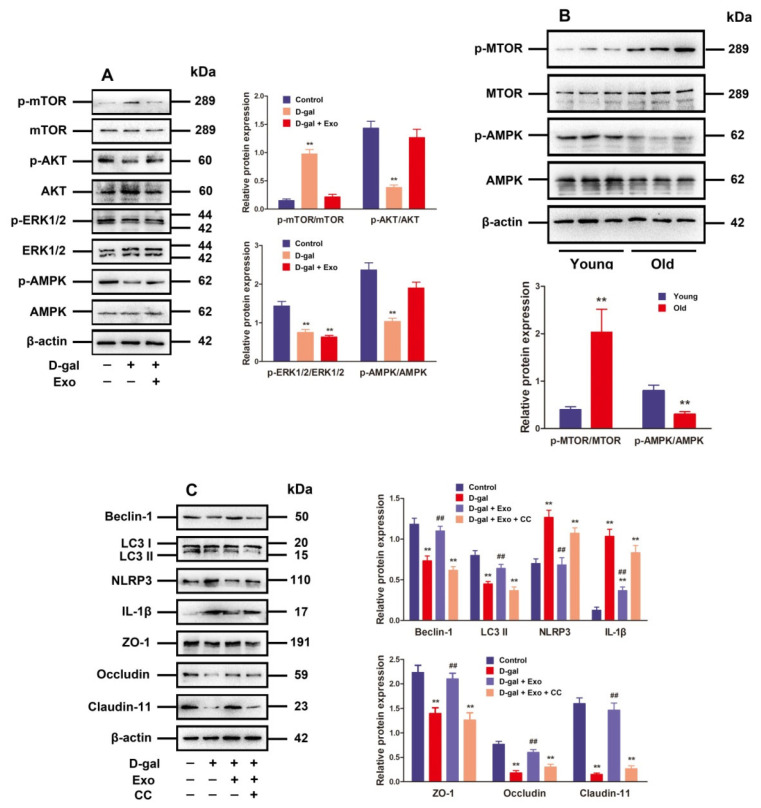
BMSC-Exos regulate autophagy and TJ function through the AMPK/mTOR signaling pathway. (**A**) SCs are subjected to D-gal (50 g/L) and/or BMSC-Exos (20 μg/mL) processing for 48 h. The protein expressions of p-mTOR, mTOR, p-AKT, AKT, p-ERK1/2, ERK1/2, p-AMPK, and AMPK are investigated using Western blotting. (**B**) Western blotting is performed to examine the relative expressions of p-mTOR, mTOR, p-AMPK, and AMPK proteins obtained from porcine testes. (**C**) SCs receive 48 h of treatment with D-gal (50 g/L), CC (10 μM), and/or BMSC-Exos (20 μg/mL). Western blotting for protein expressions of Beclin-1, LC3, NLRP3, IL-1*β*, ZO-1, Occludin, and Claudin-11 is accomplished with β-actin as the loading control and the values exhibited in the format of mean ± SD; ** *p* < 0.01 vs. the control group; ^##^ *p* < 0.01 vs. the D-gal treatment group.

The mechanistic target of rapamycin kinase (mTOR) serves as a critical negative regulatory molecule for autophagy, and mTOR phosphorylation inhibits the formation of autophagosomes [21]. Since p-mTOR is regulated by ERK1/2 and AKT activation as well as AMPK inactivation [21], ERK1/2 phosphorylation, AKT, and AMPK in D-gal-induced aging porcine SCs after treatment with BMSC-Exos were examined. According to the findings, D-gal markedly strengthened the phosphorylation of mTOR while suppressing P-ERK1/2, P-AKT, and p-AMPK expressions. Moreover, the treatment with BMSC-Exos clearly decreased p-mTOR expression but increased the expressions of P-AKT and p-AMPK in D-gal-induced aging SCs, while the p-ERK1/2 expression was not notably influenced (Figure 5A). Moreover, the raised p-mTOR level plus decreased p-AMPK level in old porcine testes were observed (Figure 5B), which was consistent with the results obtained from SCs.

In this study, CC was used to determine whether BMSC-Exos promote autophagic flux and TJ function in D-gal-induced aging SCs through the AMPK signaling pathway. The stimulatory effect of BMSC-Exos on autophagy-related markers and TJ-related proteins as well as their inhibitory effects on NLRP3 and IL-1 protein expressions were repressed by CC in D-gal-induced aging SCs (Figure 5C), demonstrating that BMSC-Exos can reduce p-mTOR by stimulating p-AMPK to maintain autophagic flux and TJ function in aging porcine SCs.

### 3.6. BMSCs and BMSC-Exos Improved Autophagy and BTB Function as Well as Inhibited ROS Generation and NLRP3 Inflammasome Activation in the Testes of D-Gal-Induced Aging Mouse Model

**Figure 6:**
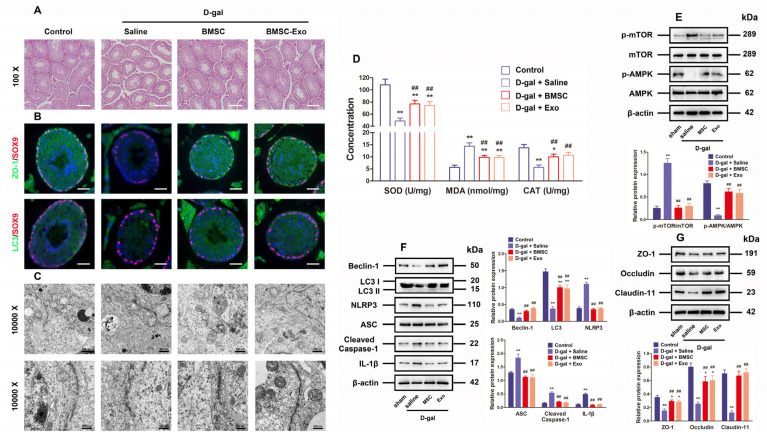
BMSCs and BMSC-Exos improve autophagy and BTB function while repressing ROS generation and NLRP3 inflammasome activation in the testes of D-gal-induced aging mouse model. (**A**) The histological changes in testes are appraised with the help of HE staining (100× for original magnification, scale bar = 200 μm). (**B**) Typical immunofluorescence images for SOX9 (red)-based localization of ZO-1 and LC3 (green) in the testes are generated (original magnification = 400×, scale bar = 50 μm). (**C**) TEM is applied to visualize the ultrastructure of autophagy and TJ in porcine SCs (10,000× indicating original magnification; scale bar = 500 nm). (**D**) Changes in SOD, MDA, and CAT content are examined. (**E**) The measurement of relative expressions of p-mTOR, mTOR, p-AMPK, and AMPK proteins in mouse testes is realized through Western blotting. (**F**) Beclin-1, LC3, NLRP3, ASC, Cleaved-Caspase-1, and IL-1β in mouse testes are detected for relative protein expressions using Western blotting. (**G**) Western blotting for determining the relative protein expressions of ZO-1, Occludin, and Claudin-11 in mouse testes is conducted by setting β-actin as the loading control and the value format of mean ± SD; * *p* < 0.05 and ** *p* < 0.01 vs. the control group; ^##^ *p* < 0.01 vs. the D-gal treatment group.

**Figure 7:**
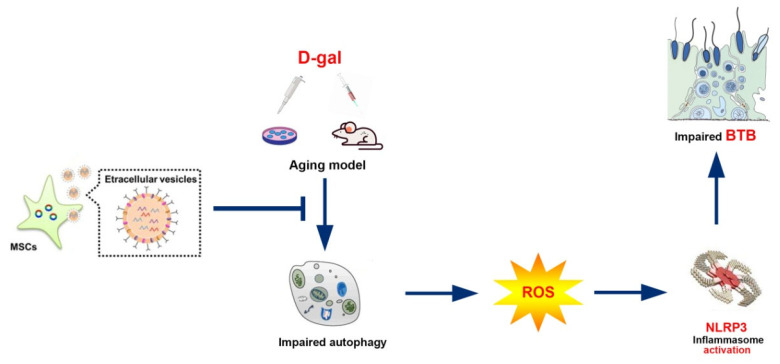
BMSC-Exos alleviate the BTB dysfunction in ageing porcine testes by activating autophagy and inhibiting ROS/NLRP3 inflammasomes through the AMPK/mTOR signaling pathway.

For the purpose of further researching BMSCs and BMSC-Exos to determine their roles in vivo, an aging mouse model was constructed by chronically injecting D-gal as reported in previous research. As revealed by histological examination through hematoxylin-eosin staining, both BMSCs and BMSC-Exos ameliorated the testicular morphology and structure as well as raised the number of layers in the testicular tubules (Figure 6A). Immunofluorescence staining for ZO-1 and LC3 demonstrated that the marker expressions regarding TJ function and autophagy were reduced in the aging testis models, but they were recovered by treatment with BMSCs and BMSC-Exos (Figure 6B). The TEM findings also uncovered that the number of autophagosomes and BTB disassembly was ameliorated by BMSC and BMSC-Exos treatment for D-gal-induced aging mouse testes (Figure 6C). The testicular CAT and SOD content also increased following BMSC and BMSC-Exos treatment, while the MDA content declined greatly in mice subjected to chronic D-gal injection (Figure 6D). Western blotting analysis further indicated that BMSCs and BMSC-Exos restored the levels of LC3, p-AMPK, Beclin-1, autophagy-related markers, as well as TJ-related proteins, Claudin-11, ZO-1, and Occludin, but reduced the levels of ASC, p-mTOR, NLRP3, Cleaved-Caspase-1, and IL-1β in the testes of aging mouse model (Figure 6E–G). In summary, data from the in vivo experiment confirm that BMSCs and BMSC-Exos can restore BTB disruption in D-gal-induced aging mice by means of AMPK/mTOR-mediated autophagy, thus restraining ROS production and NLRP3 inflammasome activation. Collectively, these results highly suggest that BMSC-Exos can virtually recover BTB function in aging porcine testes (Figure 7).

## 4. Discussion

During spermatogenesis, the developing germ cell-enclosed SCs perform the function of modulating germ cell development and spermatogenesis based on the functions of providing nutrients while participating in BTB formation. The impaired or decayed testicular function and structure emerges along with increasing age, thus disrupting the BTB, aggravating seminiferous epithelium damage, and finally resulting in spermatogenesis dysfunction and male infertility [3,4]. It was discovered through the present study that BMSC-Exos might restore the TJ function of senescent porcine SCs and the testes of aging mouse model. The underlying mechanism is that BMSC-Exos exert their protective effect by enhancing autophagy to repress ROS production and subsequent NLRP3 inflammasome activation in SCs.

Under normal physiological and pathological circumstances, autophagy in SCs has an essential effect on their survival and function [22]. It has been recognized that autophagy-related protein 5 (ATG5) and ATG7 are important players in autophagosome biogenesis. To be specific, ATG5 or ATG7 knockout in testicular SCs mitigates autophagy, thus affecting the fertility of male mice [22]. Previous studies also argued that moderate autophagy reduces the apoptosis of aging cells and improves their survival, but senescent cell damage may be aggravated by excessive autophagy [7]. As denoted by a number of other studies, the upregulated autophagy alleviates TJ dysfunction in aging-induced blood-brain barrier [23]. According to previous studies, several hallmarks of aging, including reduced longevity, worsened oxidative stress, exacerbated mitochondrial dysfunction, and decreased completion of the autophagic flux, are observed from the premature aging characteristics under induction by chronic D-gal exposure, which are similar to those of natural aging [19]. In this study, D-gal dose dependently decreased TJ function and autophagy in porcine SCs. More importantly, the autophagy inducer rapamycin blocked the D-gal-induced autophagy degradation and promoted TJ function, while the lysosomal inhibitor CQ aggravated the D-gal-induced autophagy and TJ dysfunction. These data supported the notion that insufficient autophagy exerts an upstream role in D-gal-induced TJ function in porcine SCs.

Numerous investigations have demonstrated the protective effects of MSCs on such reproductive organs as ovary and testis [15,16]. As for the testis, MSC transplant can delay testis aging and increase androgen secretion [20]. The central mechanism of such a process involves paracrine instead of MSC propagation and differentiation [17,20]. Exos have become the most explored paracrine substance over the past years, which has extensive application as a treatment strategy concerning degenerative diseases like osteoarthritis (OA) [24] and premature ovarian failure (POF) [25]. Exos functioning as lipid nanovesicles can directly penetrate the blood-brain barrier and reach the lesion sites easily [26]. It has been verified that in the case of diseased neurons undergoing toxic protein aggregate exposure, BMSCs facilitate autophagy, thereby improving neuronal survival [27]. Moreover, a very recent study elucidated that MSC-Exos can reduce the death of ischemic cardiomyocytes, which has relation to the mitigation of ischemia-induced autophagy [28]. The data from this study testified that Exos excreted by BMSCs were conducive to autophagy and TJ function in D-gal-induced senescent porcine SCs. In vivo experiment also demonstrated that the stimulatory effect of BMSC-Exos on autophagy and TJ function in the testes of aging mouse model is similar to that of BMSC transplants. These results forecast that BMSC-Exos serve as a positive regulator of TJ function and its mechanism is partly dependent on the stimulation of autophagy.

ROS, another type of pathology commonly detected in numerous infertile men, has been recognized as the key player in triggering NLRP3 inflammasome formation [13]. The NLRP3 inflammasome-related signaling pathway stimulates IL-1*β* and other pro-inflammatory mediators to start generation and subsequently accelerates the release of TNF-*α* and other pro-inflammatory cytokines, thereby leading to systemic chronic inflammation due to aging [29]. NLRP3 inflammasomes have been proven by a previous study to be activated by ROS generation, which cause D-gal-triggered learning and memory impairment in mice [30]. Moreover, it has been elaborated that the ROS generation stimulates the NLRP3 inflammasome activation to facilitate cardiocyte aging [31]. On the other hand, large quantities of investigations have confirmed the roles of MSCs or MSC-Exos in affecting ROS generation and NLRP3 inflammasome activation, including injury or inflammation [32,33]. The data of this study demonstrated that old porcine testes presented obvious increases in ROS level and NLRP3 inflammasome activation by comparison with the young ones, implying that ROS and inflammation have impacts on the old testes. Moreover, BMSC-Exos ameliorated ROS generation and NLRP3 inflammasome activation in D-gal-induced aging models were established both in vitro and *in vivo*. NAC, MCC950 (NLRP3 inhibitor), and IL-1Ra were examined to validate whether the interference of BMSC-Exos in ROS generation and NLRP3 inflammasome activation has correlation with the reversal of D-gal-induced TJ dysfunction. According to the results of this study, NAC, MCC950, and IL-1Ra could all rescue the expressions of ZO-1, Occludin, and Claudin-11 in D-gal-induced aging SCs. The above findings explicitly corroborate that the suppressed ROS production and NLRP3 inflammasome activation are involved in the rescue of TJ in senescent SCs by BMSC-Exos.

Autophagy is a crucial player in clearing ROS, misfolded proteins, proinflammatory cytokines, and ATP that trigger the activation of NLRP3 inflammasomes [34,35]. In addition, it has been manifested that the packaging and degradation of NLRP3 inflammasome components ASC and NLRP3 by autophagy-related proteins have been testified through increasing evidence [35]. It is crucial that autophagy directly eliminates mature IL-1*β* as well [36]. It was unveiled through this study that rapamycin alleviated ROS generation and NLRP3 inflammasome activation in D-gal-induced aging porcine SCs, while CQ alone aggravated and blocked the suppression of BMSC-Exos on ROS generation and NLRP3 inflammasome activation. Based on these data, BMSC-Exos have become a feasible strategy for curing D-gal-associated TJ dysfunction in SCs via autophagy promotion while repressing ROS production induced NLRP3 by inflammasome activation.

Furthermore, the AMPK/mTOR signaling pathway is known to be intimately related to the autophagy-modulated TJ function in intestinal epithelium barrier [37] or blood-brain barrier injury [38]. BMSC-Exos in this study were proven to increase the p-AMPK/AMPK ratio but decrease the p-mTOR/mTOR ratio in the in vivo and in vitro aging models. Moreover, the CC-induced AMPK inhibition exerts a reversing effect on the BMSC-Exos-mediated increase in autophagic flux and TJ function as well as the decrease in ROS production and NLRP3 inflammasome activation, suggesting that autophagy enhancement in D-gal-induced aging porcine SCs by BMSC-Exos treatment alleviates ROS production plus subsequent NLRP3 inflammasome activation to recover TJ function, which is minimally and partially realized by regulating the AMPK/mTOR signaling pathway.

There are several limitations of our current study. Firstly, it is possible that our in vivo experiments were not sufficient since we were not able to test the BMSC-Exos function in aging porcine models. A future study will examine the aging porcine model in depth, in order to reveal the administration and dosage of Exos. In addition, numerous studies have found that miRNAs or proteins in the Exos play a major role in many disease models [39,40]. Hence, the effects of individual RNA, miRNA, and related proteins in Exos need to be determined in future work, so as to validate their respective roles in the abovementioned effects.

## 5. Conclusions

In summary, it is evidenced by the present study that BMSC-Exos effectively alleviate the BTB function of ageing porcine testes by activating autophagy while inhibiting ROS/NLRP3 inflammasomes through the AMPK/mTOR signaling pathway. The obtained findings provide new insights into the therapeutic effect of BMSC-Exos on rehabilitating the senescent porcine SCs from TJ function and ameliorating the aging mouse model for BTB function.

## Data Availability

The data are available from the corresponding authors upon request.
